# Field-derived estimates of costs for Peste des Petits Ruminants vaccination in Ethiopia

**DOI:** 10.1016/j.prevetmed.2018.12.007

**Published:** 2019-02-01

**Authors:** Nicholas A. Lyons, Wudu T. Jemberu, Hassen Chaka, Jeremy S. Salt, Jonathan Rushton

**Affiliations:** a The Pirbright Institute, Ash Road, Pirbright, Woking, GU24 0NF, UK; b European Commission for the Control of Foot-and-Mouth Disease (EuFMD), Food and Agriculture Organisation of the United Nations, Rome, Italy; c University of Gondar, Ethiopia; d Food and Agriculture Organisation of the United Nations, Addis-Ababa, Ethiopia; e Global Alliance for Livestock Vaccines and Medicine, Edinburgh, UK; f Institute of Infection and Global Health, University of Liverpool, IC2 Building, 146 Brownlow Hill, Liverpool, L3 5RF, UK

**Keywords:** Peste des petits ruminants, PPR, Economics, Vaccination

## Abstract

In 2015, the OIE and FAO launched a global eradication programme for Peste des Petits Ruminants (PPR). Vaccination is a major component of this strategy yet the costs of implementing a campaign are unknown or based on assumptions without field-based verification necessary for effective economic planning. This study used experiences of attending four PPR vaccination campaigns in Ethiopia to estimate various cost components in pastoral and mixed-crop livestock systems. These components included: cost of vaccine; vaccine transport from the producer to the local storage facility; storage of vaccine at the local facility; delivery and administration of vaccine in the field; opportunity cost of farmer’s time to attend the vaccination; co-ordination of vaccination campaign; publicity and mobilisation costs; vaccine wastage from missed shots and vaccine discard. The overall cost of vaccination was approximately 6 Ethiopian birr (ETB) or US$0.2 per animal in the mixed-crop livestock system compared to approximately 3ETB or US$0.1 in pastoral areas. The relative importance of cost components varied in the two systems with farmer time being the largest contributor in the mixed-crop livestock system while field delivery was the main cost in pastoral areas. Notable vaccine wastage was observed particularly through missed shots that were typically between 0 and 10% but as high as 33%. At the national level, the output of the stochastic model showed the cost of vaccination to be highly variable particularly in the mixed-crop livestock system. These results highlight the importance of doing economic assessments of vaccination campaigns and issues that may be compromising efficiency of delivery and vaccine coverage. It is recommended that the framework be used for further economic evaluations of vaccination for PPR and other livestock diseases particularly when limited public or donor funds are being used, and that the approach be expanded to other countries and regions.

## 1 Introduction

Peste des Petits Ruminants (PPR) is a viral disease of sheep and goats associated with high morbidity and mortality in affected flocks and found in large parts of Africa and Asia (Banyard et al., 2010). In 2015, FAO and OIE launched a Global Control and Eradication Strategy for PPR which utilises a progressive stepwise approach with four stages: 1) Assessment of epidemiological situation; 2) Implementation of control activities; 3) Eradication; 4) Post-eradication (FAO and OIE, 2016). The strategy is based on vaccination, which is a key activity in stages 2 and 3. The PPR vaccine promoted by the programme stimulates longevity of antibodies post vaccination and is believed to confer immunity for at least three years, longer than the lifespan of many small ruminants (Diallo et al., 2007). Despite this immunogenicity, maintaining effective vaccination coverage will be a major challenge due to issues including: a large at risk population; high population turnover; seasonal fluctuations in animal numbers; inaccessibility of herds; high rates of animal movements; lack of co-operation from livestock owners including a reluctance to vaccinate; relatively unknown marketing systems (Albina et al., 2013). As part of the progressive control, the vaccine supply chain must also be robust and responsive to ensure vaccine availability to eliminate virus circulation. A risk-based approach to vaccination is desirable especially in the early stages of control (Singh, 2011) and is likely to be more cost effective. However, surveillance systems in affected countries are currently unable to provide sufficient understanding of PPR epidemiology in different settings to use this approach globally (Albina et al., 2013). In addition, vaccination is not synonymous with immunisation. Reasons for an animal not responding effectively to a vaccination may include: poor vaccine quality; inadequate maintenance of the cold chain; and in young animals, maternal immunity interfering in vaccine response. These issues contribute to uncertainties in the estimated cost of global control and eradication, which is made worse by the lack of reliable information on the cost of vaccination per animal in different production systems, with the economic analyses relying on assumptions without field validation. For example, when evaluating the economic impact of global eradication of PPR, Jones et al (Jones et al., 2016) used estimates based on the cost of rinderpest vaccination because the cost data for PPR vaccination in small ruminants were not available in the literature. Tago et al (Tago et al., 2017) recently adapted a costing tool used by the World Health Organisation to livestock vaccination using PPR in Senegal as an example. The approach was useful for planning resource allocation although these estimates were not derived from field observations or externally validated.

For the PPR global eradication programme to be successful, decision-making on the allocation of resources and their effectiveness could be informed by collecting data and experiences from actual vaccination programmes. In particular, cost data of implementing vaccination will be vital to identify weaknesses of vaccine distribution and delivery. This is regularly carried out in human vaccination programmes, yet has not been formalised in animal health settings (Babo Martins and Rushton, 2014). A recent review on the use of economic and social data in veterinary vaccine development revealed the relative paucity of socio-economic analyses in this field (Thomas et al., 2018). Such data are the basis for truly evidence-based benefit-cost analyses or assessing the cost-effectiveness of new technologies (Griffiths et al., 2011). Given the important gap in vaccination cost information from animal health in general, and PPR in particular, the aim of this study was to collect data from ongoing PPR vaccination campaigns in Ethiopia to provide information on the cost in different production systems and to estimate the annual cost to the Ethiopian economy. During this process, a data collection methodology was developed that could be applied in other countries and for other livestock diseases.

## 2 Materials and methods

### 2.1 The cost model

The cost of vaccination was assumed to include the following components aggregated using the model:

Vaccination cost = Vaccine cost + vaccine transport cost + Storage cost + field delivery cost + coordination cost + mobilization/ publicity cost + farmers time + vaccine wastage

Where:

Vaccine cost = vaccine price + reconstituting saline price

Vaccine transport cost = transport truck cost + fuel cost + transporting personnel cost

Storage cost = (vaccine storage freezer depreciation rate + electric power cost) * time vaccine spent in storage

Field delivery cost = field vaccination personnel cost + field transport cost (car, fuel and car maintenance cost) + Material cost (price of consumables + depreciation of durable vaccination and cold chain equipment)

Farmers’ time = hours spent by the farmers in getting their flocks vaccinated * wage per hour

Coordination cost = salary, per diem and transport cost of the local vaccination coordinator.

Mobilization cost = cost of personnel doing mobilization of stock owners + other media broad cast costs

Vaccine wastage= (vaccine cost * missed shots) + (vaccine cost * vaccine discard)

The different delivery components (i.e. transport, storage, delivery, co-ordination and mobilization) were calculated on a per dose basis with the denominator being the total number of doses used during the data collection period (i.e. including any wastage). The farmer’s time was included as an opportunity cost assuming this time could be spent doing manual labour and was also calculated based on the number of farmer’s animals vaccinated.

### 2.2 Data collection

**Fig. 1:**
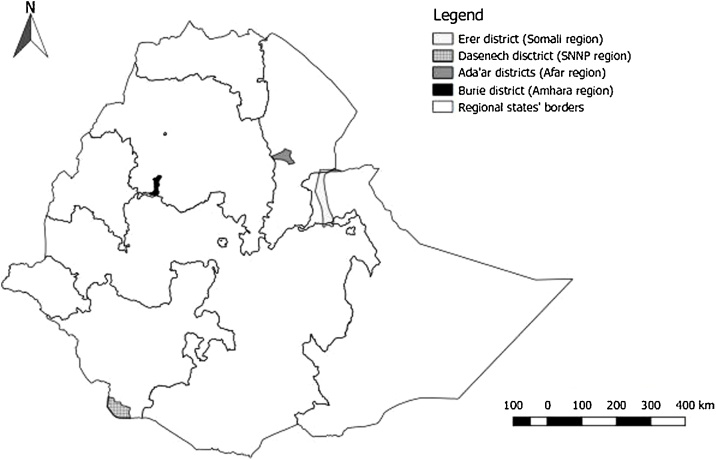
Map of Ethiopia showing the regional boundaries and districts where data were collected from PPR vaccination campaigns to estimate the cost of vaccination.

Data for the cost model were collected from vaccination conducted in four regional states in Ethiopia: Afar, Amhara, Somali and Southern Nations Nationalities and Peoples (SNNP) regional states. In each region, data were collected by one of the authors (WJ) from a single district: Ada’ar district from Afar, Erer district from Somali, Dasenech district from SNNP and Burie from Amhara (Fig. 1). The choice of region and district was based on availability of active PPR vaccination programs during the study period (February to August 2017).

The vaccinations in Afar, Somali and SNNP regions were within pastoral systems. These were implemented by the Food and Agriculture Organization of the United Nations (FAO) as part of a progressive stepwise approach against PPR in the lowlands of Ethiopia under the EU funded-project “Pursuing Pastoral Resilience through Improved Animal Health Service Delivery in Pastoral Areas of Ethiopia”. The vaccination in these regions was organized and implemented in the form of campaigns covering entire pastoral areas delineated by participatory disease search teams around an area where PPR virus was confirmed by pen-side test (Rapidtest). The vaccination in the Amhara region, which has a mixed crop-livestock system, was also reactive but in response to passive surveillance performed by individual districts using the Ethiopian government resources. In this region annual regular preventive vaccination had occasionally been delivered but not during the study period.

A data collection checklist was developed to collect data on the components of the economic cost model and it was applied through a combination of interviews and field observations in each district. Cost data on vaccine transport, vaccination coordination and mobilization were collected from interviews with vaccination coordinators. Cost data on field delivery, vaccine wastage from missed shots, and farmer’s opportunity cost from time getting their animals vaccinated were collected by personal observation of the study enumerators (animal health personnel recruited for data collection) during vaccination using a prepared format. Field observations of at least one vaccination team were carried out for three days in each district except in Bure where the observation was for only one day. During field observation, enumerators recorded the number and composition of vaccination personnel in a team and the number of animals vaccinated by the team per day. Additionally, close observations of injections from the beginning to the end of a full vaccination gun (typically 30 doses) allowed recording of the proportion of missed injections. Farmers attending the vaccination were asked the time taken to bring their animals and return to their origin including the waiting time at the vaccination site.

### 2.3 Data analysis

All field data were manually recorded and initially entered into a MS Excel spreadsheet before being transferred into RStudio (v1.0.143) for analysis. The cost for each component of the cost model was calculated and aggregated to estimate the cost per dose of vaccine used. Probability distributions for each cost component were fitted based on the collected data and were applied in the estimate of the PPR vaccination costs at population level stratified by production system (pastoral versus mixed-crop livestock). Livestock population data were available online from the Central Statistical Agency of Ethiopia (http://www.csa.gov.et/index.php/survey-report/category/348-eth-agss-2016, Appendix A). The simulation was run 100 times.

Based on observations in the field there was noticeable vaccine discard (i.e. vaccine remaining in syringes and bottles disposed when moving between vaccination sites or at the end of the day) although specific records were not kept. To account for this wastage, it was assumed that for each site visited up to ten bottles were opened and half of one of these bottles was discarded. This indicated an average of 14.6% vaccine discard. This extra cost of vaccine was added to the cost model. Since the number of bottles used, and thus the proportion of wastage is expected to vary with each site (proportion of wastage decreases exponentially as the number of bottles opened increase), this was fitted as an exponential distribution in the final model (Appendix B).

## 3 Results

**Fig. 2:**
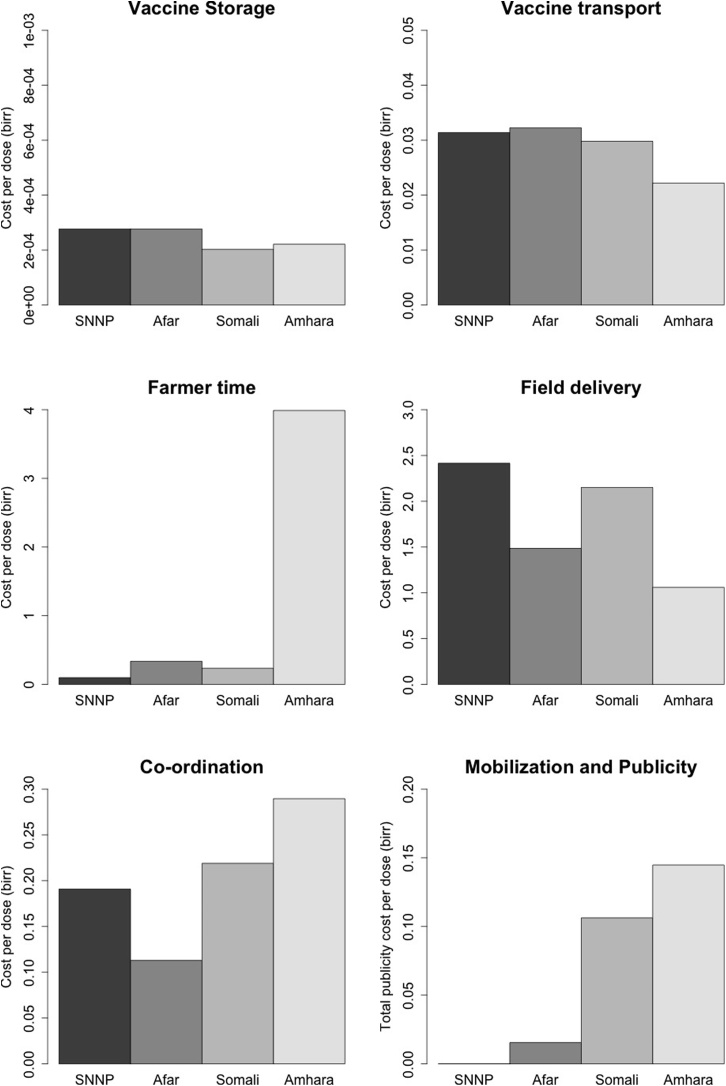
Different costs associated with different cost components in four regions of Ethiopia or PPR vaccination (all costs in Ethiopian birr, ETB).

Four vaccination campaigns were observed as part of the study. One region, Amhara, is a highland area dominated by mixed crop-livestock systems while the remaining regions are all pastoral areas. The prices of vaccine (0.49 Ethiopian birr [ETB]/dose) and reconstituting saline (0.04 ETB/dose) were the same for all regions as they were purchased from the same source, the National Veterinary Institute in Bishoftu. The costs associated with the different components of delivery for each examined region is shown in Fig. 2 with tables presented in Appendix C. Vaccine transport and storage costs were similar among the different regions but differences were seen for the other parameters most noticeably for the farmer time component in Amhara. Field delivery costs were highest in the SNNP where there was minimal expenditure on publicity and mobilisation.

**Fig. 3:**
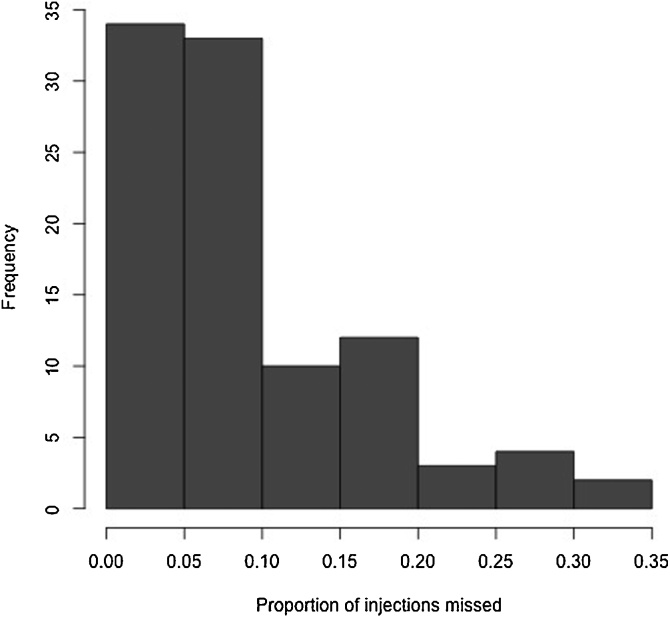
Frequency distribution of the mean proportion of PPR vaccinations missed from data collected at four different sites in Ethiopia.

**Fig. 4:**
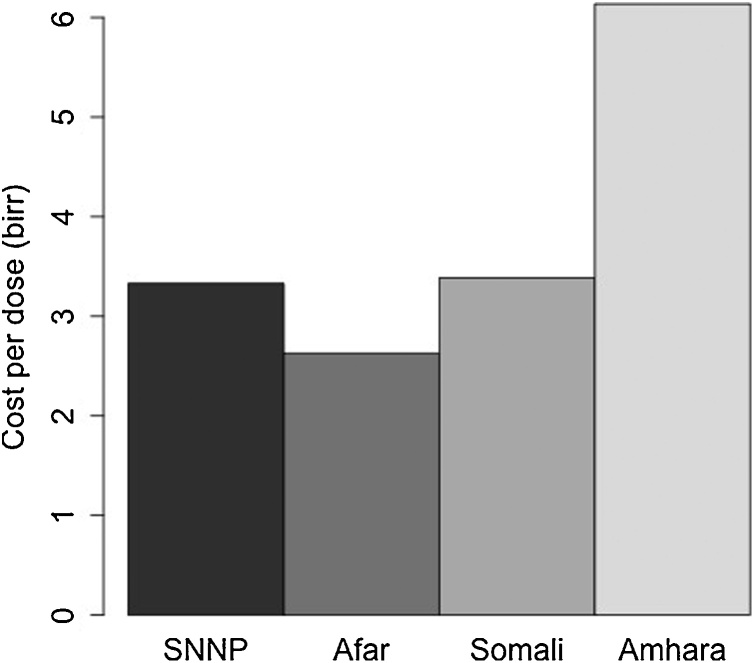
Total mean costs for each animal vaccinated for PPR in four regions of Ethiopia incorporating the cost of vaccine and delivery accounting for wastage through missed doses and discarded partially used bottles in each area (all costs in Ethiopian birr, ETB).

Observations of the vaccination in the field indicated that a proportion of the attempted vaccinations failed as the needle was not applied to the animal and the vaccine was simply ejected onto the animal’s coat or floor. Fig. 3 shows the percentage of vaccination shots that were missed due to inadequate application at the herd/flock level ranged from 0 to 33% (overall mean across all four locations was 12.2%). Fig. 4 incorporates the cost of vaccine and associated wastage through missed shots and discards. The cost for the overall vaccine cost (vaccine, delivery and opportunity costs of farmers time) was approximately 6 ETB per animal in Amhara compared to approximately 3 ETB in the other areas indicating that vaccination costs in mixed crop livestock systems are approximately double that in pastoral areas of Ethiopia. A summary of the costs per animal in the four different locations is shown in Fig. 4 with a range of just over 3 ETB per animal in SNNP and just over 6 ETB in the mixed crop livestock system in Amhara.

**Fig. 5:**
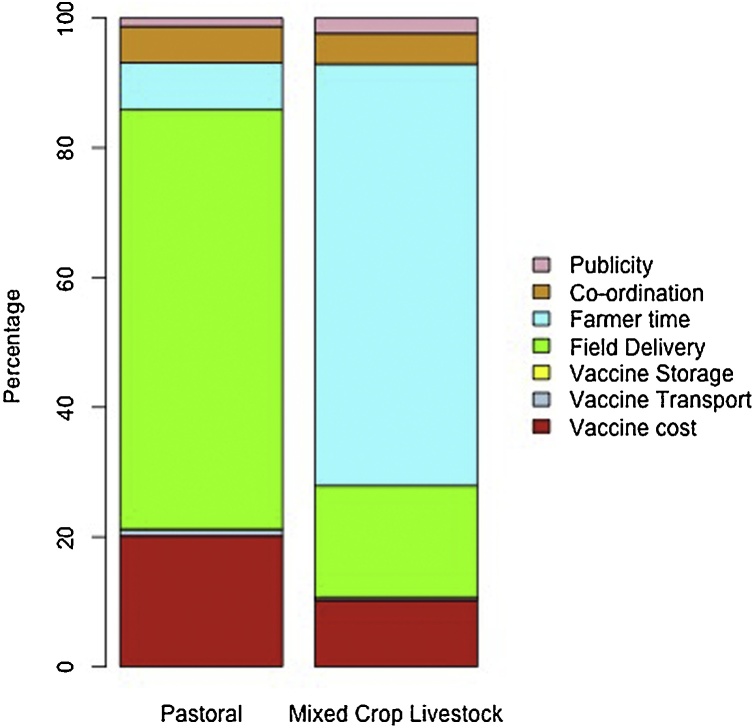
Percentage contribution of various components to PPR vaccination costs in pastoral and mixed crop livestock systems in Ethiopia.

Differences were observed in the contribution of the different cost components to the overall costs in the different systems. In pastoral areas, the main cost was the field delivery whilst farmer’s time was the largest contributor in the mixed-crop livestock system (Table 1, Fig. 5).

**Table 1:** Relative percentage of different cost components contributing to the mean vaccination cost for PPR among Pastoral and Mixed-Crop livestock systems in Ethiopia. Costs in Ethiopian birr (ETB).

Component	Mean cost (ETB) per dose (%)	
	Pastoral	Mixed Crop Livestock
Vaccine	0.53 (20.2)	0.53 (10.3)
Vaccine transport	0.031 (1.0)	0.022 (0.36)
Vaccine storage	0.00025 (0.0081)	0.00022 (0.0036)
Field delivery	2.0 (64.8)	1.1 (17.3)
Farmer’s time	0.22 (7.2)	4.0 (65.0)
Co-ordination	0.17 (5.6)	0.29 (4.7)
Publicity	0.041 (1.3)	0.14 (2.4)

**Fig. 6:**
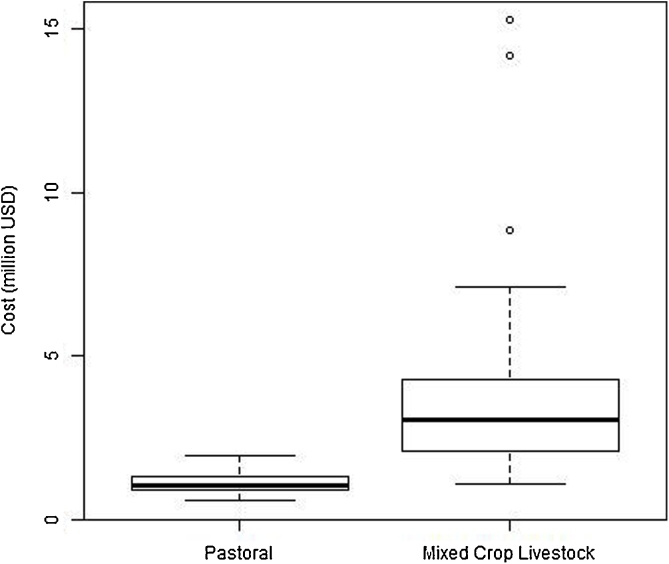
Population level costs (in $US) for PPR vaccination for a single year in Ethiopia at 80% coverage of all small ruminants.

Fig. 6 presents the national level costs based on the parameters given in Table 2. The median estimate from the simulation estimated the cost in the pastoral system as US$1.0 million (range 0.57–1.9) compared to $US3.0 million (range 1.1–15.3) in the mixed crop livestock system assuming 80% vaccination coverage in both systems in a one year period.

**Table 2:** Parameters used for population level stochastic model to estimate national PPR vaccination costs in Ethiopia based on collected field data.

Parameter	Pastoral		Mixed-crop livestock	
	Distribution	Values	Distribution	Values
Vaccine transport	Uniform	Min: 0.030 Max:0.032	–	0.022
Vaccine storage	Uniform	Min: 0.00042 Max: 0.00050	–	0.0020
Field delivery	Gamma	Rate: 7.3 Shape: 3.6	–	1.1
Farmers time	Exponential	Rate: 5.5	Exponential	Rate:0.25
Co-ordination	Uniform	Min: 0.11 Max:0.22	–	0.29
Publicity	Uniform	Min: 0 Max: 0.11	–	0.14
Missed shots	Exponential	Rate: 10.3	Exponential	Rate: 10.3
Vaccine discard	Exponential	Rate: 6.8	Exponential	Rate: 6.8
Proportion adult animals	–	0.6	–	0.6
Proportion young animals	–	0.4	–	0.4
Adult vaccine coverage	–	0.8	–	0.8
Young vaccine coverage	–	0.8	–	0.8

## 4 Discussion

The results presented are the first published account of PPR vaccine delivery costs based on field data incorporating vaccine wastage and opportunity costs for farmers. The average vaccination cost per dose was approximately 6 and 3 ETB in mixed crop-livestock and pastoral systems respectively. Based on the exchange rate at the time of writing, this equates to around 0.2 and 0.1 US$ respectively. An unpublished rapid evaluation of costs by consultants estimated values of 1.13 (0.04US$) and 2.12 ETB (0.078 US$) in the pastoral Somali region when performed by private and public veterinary services respectively. Unlike the results in this study, these latter estimates did not consider the impact of opportunity cost of farmer’s time, vaccine storage, or the cost of the vaccine (incorporating vaccine wastage through missed shots or residual wastage in vials), which likely accounts for the lower observed costs.

The estimates from Tago et al (Tago et al., 2017) based on a model parameterised with data from Senegal ranged from 0.20 USD (95%CI 0.18-0.21) to 0.34USD (95%CI 0.24-0.54) per dose depending on the assumptions made on number of animals vaccinated per day. These estimates are similar to those reported in this study although they did not consider the opportunity costs of the farmers and the model was mostly parameterised from expert opinion. In their model, they highlighted the importance of team “productivity” in terms of numbers of animals vaccinated per day. The assumed number of doses administered per team was lower than that recorded in the current study, even in the high productivity scenario. Related to vaccinator productivity, it is worth highlighting that vaccination is not the same as immunization, and a better evaluation would be based on serological outcomes rather than simply numbers of animals vaccinated.

The greatest proportion of the cost varied in the different production systems. In the pastoral system it was field delivery, while in the mixed crop-livestock system it was farmer’s time. Tago et al (Tago et al., 2017) also reported the relative contribution of different cost components. Under high productivity scenarios the largest contributor was “injection supplies” followed closely by the cost of vaccine and staff. In the low productivity scenarios, staff time was over half of the cost. Farmer time was not considered in their analysis but has important implications as it represents an opportunity cost in terms of revenue forgone by the farmer in participating in the vaccination campaign. This component differed greatly in the production systems at approximately 65% in the mixed crop livestock system and 6% in the pastoral system. The actual time invested by farmers was equivalent in the two systems but the smaller sizes of flocks/herds in the mixed crop livestock system means the farmer has to spend relatively more time per animal owned. Flock/herd sizes tend to be lower in mixed crop livestock systems so this aspect could potentially compromise participating in vaccination campaigns aimed at high coverage to reduce the impact and circulation of PPR virus. The cost of farmer’s time per hour was based on the lowest possible value of labour. This cost is expected to vary with season and may represent an over or underestimate depending on the seasonal demands in farming. Collecting data to inform this parameter would be useful in future studies on livestock vaccination as it helps to understand which people will be most inconvenienced in the short term by vaccination campaigns.

A major objective of this work was to develop a framework for collecting data from vaccination campaigns that could be applied elsewhere and adapted for other livestock diseases. Data collection was not standardised at the beginning of the project leading to limitations in the work. These include inconsistent measurements of vaccine wastage through missed shots and insufficient records of disposal of residual vaccine when moving between sites. These limitations highlight areas that should be accounted for with greater certainty in future economic studies. However, the detailed observations made in Afar and SNNP raises significant concerns over vaccine wastage that could have an important impact on national and global cost estimates for PPR vaccination. The unexpectedly high rate of missed shots is likely related to a lack of handling facilities that were not present in any campaign area. Vaccination was delivered either into the neck or medial aspect of the thigh by lifting of the hind limbs. These wastages could be reduced with: different application methods; improved training of staff; and access to adequate equipment for vaccination and animal handling. The improved effectiveness for these different interventions needs to be tested and costed in order to see what is the more appropriate. Moreover the vial size of 100 ml at 1 ml per dose could be leading to significant wastage in systems with small herd sizes (Albina et al., 2013). Experience during the rinderpest campaign was that a lower number of doses per vial did not introduce a cost saving due to the relatively higher packaging costs per dose (Gijs Van ‘t Klooster, Personal Communication) although an economic analysis based on small ruminant systems would be worthwhile.

The present study did not attempt to assess other negative impacts of vaccination. Anecdotally, farmers report adverse events such as abortion although this has not been quantified. Such adverse events could have an impact on coverage which again is more likely in smaller flocks/herds where farmers may be less inclined to take a risk with small numbers of animals, and there is also likely a seasonal effect to the perceived risk. Vaccination campaigns should be followed with more rigorous evaluation of adverse events to understand their impact, how they may be mitigated, and the effect they may have on coverage in subsequent vaccination campaigns.

PPR is a highly transmissible infection and consequently high vaccine coverage is required to control disease at a population level. This study did not attempt to quantify the cost of an entire campaign that will include factors such as background epidemiological studies to identify high-risk areas for vaccination, post-vaccination monitoring and surveillance to evaluate the impact of the campaign. Although the vaccine is thought to provide life-long protection, the high turnover of the population creates a unique challenge for maintaining vaccination coverage that is likely to be much greater than for rinderpest with different costs involved. This study developed a framework for estimating costs of vaccination campaigns for a disease identified by OIE and FAO for global eradication. Data collection and analysis identified important factors that could be having a significant impact on vaccination costs and willingness of farmers to participate. Further studies are required based on the established framework which should be incorporated into all publically funded vaccination campaigns to ensure efficient allocation of limited resources.
