# Supplementary material for: Field-derived estimates of costs for Peste des Petits Ruminants vaccination in Ethiopia
Source: Prev Vet Med. 2019 Feb 1;163:37–43. doi: 10.1016/j.prevetmed.2018.12.007 (PMC6351750; doi:10.1016/j.prevetmed.2018.12.007)
Supplement: Supplementary file 1 [file mmc1.docx]

Appendix A

Sheep and goat population for 2016 by region and production system (from (http://www.csa.gov.et/index.php/survey-report/category/348-eth-agss-2016).

| **Region** | **Pastoral**^c^ | | **Mixed crop livestock** | |
| --- | --- | --- | --- | --- |
|  | **Sheep** | **Goats** | **Sheep** | **Goats** |
| Tigray | 0 | 0 | 2041731 | 4584138 |
| Afar^a^ | 4322898 | 8653150 | 0 | 0 |
| Amhara | 0 | 0 | 10735926 | 6438600 |
| Oromia^b^ | 1047347 | 1295453 | 8818825 | 6834331 |
| Somali^c^ | 3066894 | 4558806 | 0 | 0 |
| Benshangul-Gumuz | 0 | 0 | 100013 | 431216 |
| SNNP^d^ | 1738802 | 2983617 | 3348205 | 2274633 |
| Gambela | 0 | 0 | 44302 | 99018 |
| Harari | 0 | 0 | 7100 | 72555 |
| Dire Dawa | 0 | 0 | 64234 | 205804 |

^a^ estimate from reference is for two administrative zones. Value in this table is adjusted to reflect all five zones assuming equal numbers within each zone.

^b^ Assumes 2 administrative zones (Guji and Borena) are pastoral with remaining MCL

^c^ estimate from reference is for three administrative zones. Value in this table is adjusted to reflect all nine zones assuming equal numbers within each zone.

^d^ Assumes 2 administrative zones (Benchi Magi and South Omo) are pastoral with remaining MCL
