# Supplementary material for: Field-derived estimates of costs for Peste des Petits Ruminants vaccination in Ethiopia
Source: Prev Vet Med. 2019 Feb 1;163:37–43. doi: 10.1016/j.prevetmed.2018.12.007 (PMC6351750; doi:10.1016/j.prevetmed.2018.12.007)
Supplement: Supplementary file 2 [file mmc2.docx]

Appendix B

Assumed vaccine discard based on discarding 50% of the last bottle used for each site.

| Number of bottles used | % discard |
| --- | --- |
| 1 | 50% |
| 2 | 25% |
| 3 | 17% |
| 4 | 13% |
| 5 | 10% |
| 6 | 8% |
| 7 | 7% |
| 8 | 6% |
| 9 | 6% |
| 10 | 5% |
