# Supplementary material for: Field-derived estimates of costs for Peste des Petits Ruminants vaccination in Ethiopia
Source: Prev Vet Med. 2019 Feb 1;163:37–43. doi: 10.1016/j.prevetmed.2018.12.007 (PMC6351750; doi:10.1016/j.prevetmed.2018.12.007)
Supplement: Supplementary file 3 [file mmc3.docx]

Appendix C

Data collected from each of four study sites on the various components in the cost model. SNNP = Southern Nations, Nationalities, and Peoples' Region. ETB = Ethiopian birr.

a) Vaccine transport from vaccine producer to local field station.

| Region | Doses transported | Time taken (days) | Vehicle hire (ETB) | Fuel costs (ETB) | Daily salary ^a^(ETB) | Per diem (ETB) | Transport cost/dose (ETB) |
| --- | --- | --- | --- | --- | --- | --- | --- |
|  | a | b | c | d | e | f | b*(c+e+f)+d/a |
| Afar | 600,000 | 5 | 2500 | 4,420 | 186.1 | 300 | 0.032 |
| Amhara ^b^ | 150,000 | 5 | 2000 | 4,680 | 186.1 | 206 | 0.022 |
| SNNP | 600,000 | 5 | 2500 | 2,980 | 367.9 | 300 | 0.031 |
| Somali | 600,000 | 5 | 2500 | 2,947 | 186.1 | 300 | 0.030 |

^a^based on monthly salary of 11,037 in SNNP and 5,583 in other locations ^b^ overall estimate divided by 5 as was included with four other vaccines

b) Vaccine storage

| Location | Doses stored | Freezer price | Period of use (days) | Freezer depreciation (%) | Electricity costs (per day)^b^ | Storage cost/dose (ETB) |
| --- | --- | --- | --- | --- | --- | --- |
|  | a | b | c | d | e | (d*c*b)+(e*c)/a |
| Afar | 600,000 | 18,000 | 60 | 1.36x10^-4^ | 0.3 | 5.0x10^-4^ |
| Amhara ^a^ | 150,000 | 18,000 | 60 | 1.36x10^-4^ | 0.3 | 4.0x10^-4^ |
| SNNP | 600,000 | 18,000 | 60 | 1.36x10^-4^ | 0.3 | 5.0x10^-4^ |
| Somali | 600,000 | 12,260 | 60 | 1.36x10^-4^ | 0.3 | 4.2x10^-4^ |

^a^ overall estimate divided by 5 as was included with four other vaccines

^b^ based on an electricity cost of 0.3 ETB/kwh, energy consumption of 1 kwh per litre freezer capacity per year, and a 400 litre capacity (i.e. 0.3 x 1 x 400/365=0.3/day)

c) Field delivery including equipment (mean used for data collected for more than one day or team).

| Location | Number of teams monitored | Number of days for data collection | Doses administered per day per team | Vehicle use (days) | Vehicle hire (ETB per day) | Fuel cost (ETB) | Per diems (ETB per day) | Salary (ETB per day) | Needle costs (ETB/day) | Vaccination gun costs (ETB/day)^a^ | Cool box^b^ | Field delivery cost/ dose (ETB) |
| --- | --- | --- | --- | --- | --- | --- | --- | --- | --- | --- | --- | --- |
|  |  |  | a | b | c | d | e | f | g | h | i | (b*c)+d+f+g+h+i/a |
| Afar | 1 | 3 | 2133 | 0.367 | 2000 | 960 | 750 | 650 | 14 | 36 | 1.30 | 1.49 |
| Amhara | 1 | 1 | 600 | 1 | 200 | 54 | 100 | 200 | 14 | 67.5 | 0.32 | 1.06 |
| SNNP | 1 | 3 | 1160 | 0.33 | 2000 | 995 | 730 | 408.5 | 7 | - | 1.30 | 2.42 |
| Somali | 3 | 3 | 2444 | 1 | 2000 | 569 | 1275 | 537.6 | 84 | 30 | 1.30 | 2.15 |

^a^ based on estimated frequency of replacement of a gun worth 450 ETB

^b^ based on number of 120 ETB cooler boxes used with an estimated 0.27% depreciation per day (i.e. a cool box will last for on average a one year period.

d) Farmer’s time (hours) and associated opportunity cost

| Location | Number of sites | Number of owners interviewed | Total time | | | Number of shoats | | | Labour cost (ETB per hour) | Farmers time cost (ETB/dose) |
| --- | --- | --- | --- | --- | --- | --- | --- | --- | --- | --- |
|  |  |  | Mean | Median | Range | Mean | Median | Range |  |  |
| Afar | 3 | 31 | 3.3 | 3 | 1-6 | 111.8 | 100 | 35-300 | 10 | 0.34 |
| Amhara^a^ | 1 | 29 | 2 | N/A | 0.5-4.0 | 7.0 | 7 | 1-14 | 9.6 | 4.0 |
| SNNP | 6 | 59 | 0.44 | 0.5 | 0.25-1.0 | 43.3 | 40 | 20-83 | 8.75 | 0.098 |
| Somali | 3 | 9 | 1.7 | 1.7 | 1.5-2.0 | 96.0 | 98 | 65-125 | 12.5 | 0.24 |

^a^ travel times not recorded for individual farmers but based on discussions with seven farmers that attended the vaccination

e) Co-ordination costs (for each campaign this involved one person).

| Location | Number of doses in campaign | Length of campaign period (days) | Per diem (ETB) | Daily salary (ETB) | Co-ordination cost/dose |
| --- | --- | --- | --- | --- | --- |
|  | a | b | c | d | b*(c+d)/a |
| Afar | 126,000 | 13 | 300 | 247^b^ | 0.11 |
| Amhara | 6,000 | 5 | 100 | 247 ^b^ | 0.29 |
| SNNP | 35,000 | 10 | 300 | 368^a^ | 0.19 |
| Somali | 37,500 | 15 | 300 | 247 ^b^ | 0.22 |

^a^ based on a monthly salary of 11,037 ETB

^b^ based on a monthly salary of 7,424 ETB

f) Mobilisation and publicity costs

| Location | Number of doses in campaign | Time spent on publicity (days) | Per diem (ETB) | Daily salary (ETB) | Other costs | Co-ordination cost/dose |
| --- | --- | --- | --- | --- | --- | --- |
|  | a | b | c | d | e | b*(c+d)+e/a |
| Afar | 126,000 | 13 | 150 | 0 | 0 | 0.015 |
| Amhara | 6,000 | 2.5 | 100 | 247 | 0 | 0.14 |
| SNNP | 35,000 | 10 | 0^a^ | 0 | 0 | 0 |
| Somali | 37,500 | 15 | 262.5^b^ | 0 | 48^c^ | 0.11 |

^a^ vaccination team does publicity at a night meeting (“nab”) with no additional payments

^b^ Sometimes two kebele chairman used at 175 per diem each so midpoint between 175 and 350 ETB used.

^c^ Loudspeaker battery

g) Missed shots from observations made at three sites in Ethiopia.

| Location | Sites | Flocks | Number of injections attempted | Number missed | % missed | Mean missed per flock (range) |
| --- | --- | --- | --- | --- | --- | --- |
| Afar | 3 | 29 | 653 | 109 | 16.7 | 16.1 (0.0-30.0) |
| Amhara | - | - | Not recorded | Not recorded | 13.0^a^ | Not recorded |
| SNNP | 6 | 59 | 2521 | 146 | 5.8 | 5.6 (0.0-13.1) |
| Somali | 3 | Not recorded | 22000^b^ | 2913 | 13.2^b^ | 15.6 (0.02-33.0)^c^ |

^a^ estimated based on general observations combined with interviewing vaccinators. Individual records of missed shots not kept.

^b^ based on estimated total number of injections over a three day period by all vaccination teams in area. Teams were asked to give reports on missed shots that form the number missed in this region

^c^ individual flock data not available. Figure refers to total flocks observed per vaccination team per day
